# Quantum annealing for microstructure equilibration with long-range elastic interactions

**DOI:** 10.1038/s41598-023-33232-w

**Published:** 2023-04-13

**Authors:** Roland Sandt, Yann Le Bouar, Robert Spatschek

**Affiliations:** 1 Structure and Function of Materials, Institute of Energy and Climate Research, Forschungszentrum Jülich GmbH, 52425 Jülich, Germany; 2 Université Paris-Saclay, ONERA, CNRS, Laboratoire d’Etude des Microstructures, 92320 Châtillon, France; 3 JARA-ENERGY, 52425 Jülich, Germany

**Keywords:** Mechanical properties, Computational methods

## Abstract

We demonstrate the use and benefits of quantum annealing approaches for the determination of equilibrated microstructures in shape memory alloys and other materials with long-range elastic interaction between coherent grains and their different martensite variants and phases. After a one dimensional illustration of the general approach, which requires to formulate the energy of the system in terms of an Ising Hamiltonian, we use distant dependent elastic interactions between grains to predict the variant selection for different transformation eigenstrains. The results and performance of the computations are compared to classical algorithms, demonstrating that the new approach can lead to a significant acceleration of the simulations. Beyond a discretization using simple cuboidal elements, also a direct representation of arbitrary microstructures is possible, allowing fast simulations with currently up to several thousand grains.

## Introduction

The modeling of microstructures is an important approach to the understanding, improvement and development of new materials for various applications. However, as mechanisms at different length and time scales are intimately linked, such descriptions and model implementations are typically challenging and require massive computational resources. Although phase field simulation approaches – the most prominent method for predicting microstructure evolution – strongly benefit from developments like the thin interface limit^1,2^, nondiagonal phase field models^3,4^ and sharp phase field approaches^5^, simulations containing large microstructural domains to obtain predictions with a certain statistical significance are rare, strongly limited by the available (super-)computer resources and their associated costs and energy consumption. Despite the enormous progress in this research field and the extended use of parallel computers and graphics cards for the simulations, limitations of the computational techniques remain a serious thread to the basic scientific progress and applied research.

One of the striking questions, which arises at the horizon of materials science modeling is how quantum computing will potentially change the simulation landscape in the future. However, at present a general-purpose quantum computer of sufficient size is not yet available. In the meantime, a technology known as quantum annealing (QA)^6–10^ has emerged and is available on several sites worldwide. The use of such machines differs significantly from traditional gate based computers and therefore currently only specific problems can be handled by quantum annealers^11^. The concept of a quantum annealer is that its qubits are initialized in a well defined state which is described by a Hamiltonian with a unique ground state^12^. During the operation at cryogenic temperatures, this Hamiltonian is changed adiabatically such that the ground state converts into the one of the final, desired Hamiltonian^12,13^, and therefore allows to perform global energy minimization computations efficiently. The structure of these Hamiltonians is a binary quadratic model, which can be expressed in terms of a quadratic unconstrained binary optimization or equivalently through an Ising model^11^. Due to this specific structure, so far, materials science related applications of this technology are still rare. Instead, actual research focuses mainly on the benchmarking and performance tests of quantum annealing compared to classical approaches^14–16^.

Some first applications in the field of biology and traffic research in the sense of optimization problems have been developed recently. Here, quantum annealing enables the efficient analysis of transcription factors in gene expression with combined machine learning algorithms^17^, identification of conformations of lattice protein models^18^ and their folding^19^, detection of tree cover in aerial images^20^, real-world traffic flow optimization problems^21^ or control of automated guided vehicles^22^. However, the usage of quantum annealing in materials science is not widespread and few publications correspond to phase transitions in the transverse field Ising model^23^, the investigation of critical phenomena in frustrated magnets via the Shastry-Sutherland Ising model^24^, Monte-Carlo sampling^25^ and the automated materials design of metamaterials^26^. The purpose of the present paper is therefore to demonstrate that this novel technology can indeed lead to completely new possibilities beyond the existing and commonly used descriptions for the modeling of microstructures.

In order to be as explicit and illustrative as possible, we demonstrate here the case of coherent solid state transformations involving austenitic and martensitic phases, where the latter are allowed to appear in different variants. Such transitions play a role for shape memory alloys like NiTi, which can be deformed easily at low temperatures, but heating to higher temperatures lets the material return to its previous, trained shape^27^. The modelling and mapping of shape memory alloys to spin glass systems was previously established in several studies^28–31^ and can here be exploited for QA applications. In the following we will mainly stick to the terminology of the shape memory alloys but emphasize that similar approaches can be used to model e.g. the transformation and deformation behavior in steels, ferroelastic materials, as well as phase changes in solid electrolytes for rechargeable batteries. The particular aspect that plays a central role here are the anisotropic long-range elastic interactions, which are common for solid state transformations^32^, and therefore the ground state configuration does not only depend on phase concentrations and fractions, but also on the detailed microstructural arrangement of phases and grains. In a typical phase field simulation^33^, the microstructural evolution is solved together with the relaxation of the mechanical deformations in the spirit of a continuum description, which leads to very long simulation times. Here, we show that the separation of the discrete degrees of freedom for the variant distribution of martensitic phases from the continuous development of the microstructure and the QA treatment allow to drastically increase the performance of the computations and therefore to simulate significantly larger, application relevant systems compared to existing approaches.

## Results

### One-dimensional model

**Figure 1:**
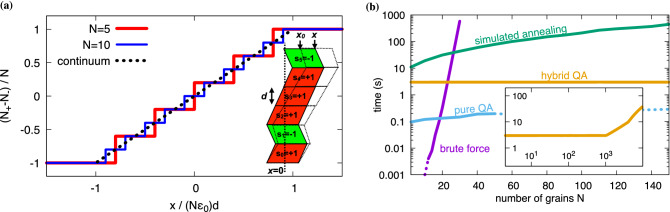
Results of the one-dimensional model comparing different numerical and analytical methods. (**a**) Mean variant orientation $(N_+-N_-) / N$ as function of the displacement $x / d N \varepsilon _0$. Comparison between the results obtained by numerical minimization (solid lines) versus the analytical theory for an infinite and continuous system (dashed line). For large displacement, all “spins” are aligned and therefore the “magnetization” saturates. The inset shows a sketch of the one-dimensional arrangement of martensite variants $s_i=+1$ (red) and $s_i=-1$ (green). The bottom row is fixed to position $x=0$, whereas the top grain has a mean position $x_0$ in the stress free state. If an additional external stress or strain is applied, the top layer is moved to position *x*, and the entire microstructure is sheared to the dashed configuration. (**b**) Elapsed computation time as a function of the number of grains. Different methods and algorithms are compared. Dashed parts of the QA curve belong to the regime of chain breaks. For large system sizes, only the hybrid quantum annealing approach remains feasible, showing an almost constant computing time need for less than 1000 spin variables (inset).

For a simplified 1D model we consider only a “martensitic” phase which is assumed to exist in two different variants. Hence the microstructure consists of a line of grains of these variants, as depicted in the inset of Fig. 1a. To be explicit, we assume that both variants have a stress free strain (eigenstrain), which leads to a shear deformation relative to the austenitic mother phase, and denote these variants by state variables $s_i=\pm 1$. As in the end we will map the description to a one-dimensional Ising model, we also use here the terminology of “spins” with two possible alignments in the spirit of a magnetic model. As each of the variants leads to a shearing of the cell, we get an overall stress free deformation of this line (compared to the shear strain free austenite), depending on the spin configuration. We assume that all grains have the same height $d$, the same elastic constants, and opposite shear eigenstrain $\pm \varepsilon _0$. As one can readily see from the inset of Fig. 1a, the stress free equilibrium position of the top grain $x_0$ depends only on the number of variants $N_+$ with orientation $s_i=+1$ and $N_-$ with $s_i=-1$, but not on the individual arrangement, which is a particularity of the simplified 1D model and the chosen eigenstrain. Hence, for a fixed number $N=N_++N_-$ spins in a row, the macroscopic stress free strain is $\bar{\varepsilon } = (N_+ - N_-)\varepsilon _0/N$, which leads to $x_0 = N d \bar{\varepsilon }$. If an external deformation is enforced, i.e. $x\ne x_0$ the elastic energy is $F_{el}=\mu _\text{eff} (x-x_0)^2$ with an effective shear modulus $\mu _\text{eff}$. Obviously, the elastic energy is minimized if the spin configuration is such that $x=x_0$, which implies $(N_+-N_-)_\text{min} = x/\varepsilon _0 d$, up to the point of saturation, where all spins are aligned. This expression serves as reference for the comparison with the numerical minimization approaches below. We note that we neglected at this stage the discrete nature of the variants, which means that the integer value $N_+-N_-$ should be as close as possible to the continuum value $(N_+-N_-)_\text{min}$ above. Although the energy in the simple 1D model does not depend on the arrangement of the variants but only on the total numbers $N_+$ and $N_-=N-N_+$, we formulate the model here on the level of the individual “spins” $s_i$ for the later extension towards higher dimensions and the use of the quantum annealer. Hence we get $N_+ - N_- = \sum _i s_i$. Inserting this into the elastic energy expression yields $F_\text{el} = \mu _\text{eff} \varepsilon _0^2d ^2 \sum _{i,j} s_i s_j - 2\mu _\text{eff} x \varepsilon _0 d \sum _i s_i +\mu _\text{eff} x^2$, where the summations run over all spins. For the implementation on a quantum annealer, we need to bring this to the Ising form of a Hamiltonian *H* with

1 $$\begin{aligned} H =\sum _ih_is_i + \sum _{i<j} J_{ij}s_is_j + H_0, \end{aligned}$$

where the first term corresponds to the interaction with an external magnetic field $h_i$ and the second term to a spin-spin interaction, which favors ferromagnetic (antiferromagnetic) ordering in case that the coupling constant $J_{ij}$ is negative (positive). The last, spin-independent term $H_0$ is only an irrelevant additive constant. From the comparison of the two above expressions we identify $h_i = - 2\mu _\text{eff} x \varepsilon _0 d$ and $J_{ij} = 2 \mu _\text{eff} \varepsilon _0^2 d ^2$. First, we note that the external deformation is here analogous to the magnetic field in the Ising description. Second, the spin-spin interaction term $J_{ij}$ is positive, hence favoring “antiferromagnetic ordering”. Also, this term is independent of the spin numbers *i*, *j*, which means that this interaction does not depend on the distance between the grains. In other words, the elastic interaction depends only on the averaged “magnetization” $N_+-N_-$, which implies a mean field interaction.

The goal of the formulation is to minimize the elastic energy and to find the optimal spin or variant configuration $\{s_i\}$. To this end, we use three different numerical approaches (see methods section), and the results are compared to the analytical solution above: First, a brute force approach iterates over all spin configurations to find the energetic minimum exactly, second we use simulated annealing as probabilistic ground state finder, and finally the quantum annealing approach. Fig. 1a shows the resulting “magnetization” $(N_+-N_-)/N$, i.e. the average variant orientation, as function of applied displacement $x/d N\varepsilon _0$, which corresponds to the magnetic field in the Ising model.

As expected, the results agree with the analytical theory up to the aforementioned discretization effect, which becomes less pronounced for large grain numbers. For high displacements saturation sets in when all variants are de-twinned, which means that all spins are either in the state $+1$ or $-1$. We note that for the investigated number of spins all used algorithms lead to the same energy minimum, which confirms that also the probabilistic approaches indeed find the global minimum states.

Fig. 1b shows the required computation time for the different methods and algorithms as a function of the number of grains *N*. All conventional algorithm implementations are based on single core computations without parallelization and are mainly shown for a qualitative comparison, as the focus of the investigations is on the quantum annealing approach. For the latter, we use quantum processing unit (QPU) implementations up to the highest possible number of spins (typically $N\approx 170$ for the Pegasus architecture^34^ of a D-Wave machine). The brute force approach, where iterations over all spin configurations are run, has the highest computation time. Even at small spin systems of around $N\approx 40$ the elapsed user time was too large for practical applications due to the simulation time scaling $\sim {{\mathcal {O}}}(2^N)$. The pure quantum annealing method produces the fastest results and ends up with an almost constant elapsed QPU access time. Overall, the computations for $N\approx 150$ are roughly three orders of magnitude faster than for the other classical approaches. Beyond around $N\approx 50$ spins, so called chain breaks^35^ occur occasionally. They result from the need to encode strongly coupled spins as a single logical spin. Ideally, these spins should represent the same state as the individual spins, but in practise this identity can be violated. To avoid this issue and to simulate even larger systems, which are essential for higher dimensional modeling in the following sections, hybrid classical and quantum annealing approaches can be used, which combine pure QA with conventional minimization approaches^36^. The numerical results in Fig. 1b show an increase of the computation time of the hybrid solver compared to the pure QA, but the relative acceleration compared to the classical algorithms becomes even more striking. For the hybrid solver, the elapsed computation time is essentially independent of the number of spin variables and increases only beyond $10^3$ grains to several seconds. Altogether, the hybrid QA is clearly the fastest approach for large grain numbers and is therefore used in the following two-dimensional simulations.

### Transformations in higher dimensions

For the determination of the linear elastic energy beyond one dimension, we consider coherent precipitates of different variants which form inside the matrix. In this way, the entire material can be considered to consist of small entities (in the following denoted as grains), which can be in one of the different martensitic states. The simplest possible (cartesian) discretization is to use small cuboidal grains with edge length *a*. All grains are assumed to be coherent (the elastic displacements and tractions are continuous at the interfaces between the grains), and we use homogeneous elasticity, i.e. we ignore differences in the elastic constants between the different phases or variants. This has the consequence that the elastic energy reduces to combinations of pairwise interactions between all grains^37^.

For demonstrational purposes we perform here two-dimensional simulations in a plane strain setup, but a transfer to three dimensions is straightforward. In particular, the annealer part does not depend on the dimensionality of the description. The qualitatively new aspect beyond 1D is the appearance of distance and orientation dependent “spin-spin” interactions, which decay only slowly with the distance between the grains, and therefore leads to fully populated matrices $J_{ij}$. As it turns out that an accurate determination of the elastic interaction energy is essential for a precise prediction of the equilibrium microstructure, we use Fourier transformation approaches with periodic boundary conditions as outlined in the methods section. As boundary conditions, we use either vanishing average stress in the periodic volume *V*, $\langle \sigma _{ij} \rangle = \frac{1}{V} \int \sigma _{ij}(\textbf{r})\,d\textbf{r} = 0$, or, similarly to the 1D description, a given average strain $\langle \varepsilon _{ij} \rangle$. We employ in the following for simplicity isotropic elasticity, which is e.g. described by the Lamé coefficient $\lambda$ and the shear modulus $\mu$, i.e. the stress-strain relationship reads $\sigma _{ij} = 2\mu (\varepsilon _{ij}-\varepsilon _{ij}^{(0)}) + \lambda \delta _{ij} (\varepsilon _{kk}-\varepsilon _{kk}^{(0)})$, where implicit summation over repeated indices is used. The position dependent eigenstrain $\varepsilon _{ij}^{(0)}(\textbf{r})$ is known for a given microstructure with fixed phase dependent stress free strains (relative to the austenitic mother phase), $\varepsilon _{ij}^{(0)}(\textbf{r}) = \theta (\textbf{r}) \varepsilon _{ij}^0$, where the indicator function $\theta$ is zero in the austenite and either $+1$ or $-1$ in the two considered martensite variants. For a given microstructure, the elastic energy can then be computed in reciprocal space, as shown in the methods section. For the formulation as Ising model we discretize our microstructure using small non-overlapping cuboidal grains as discussed above and assign a “spin” $s_i$ to each of them like before, such that the indicator field becomes a superposition $\theta (\textbf{r}) = \sum _i s_i \theta _i(\textbf{r})$, where $\theta _i$ equals one inside the corresponding square and is zero outside. Therefore, the elastic energy decomposes into pairwise interactions (for $i\ne j$) and self-energy terms (for $i=j$)

2 $$\begin{aligned} E_{i,j} = s_i s_j \frac{1}{2V} \int d\textbf{r} \int d\mathbf {r'} B(\textbf{r}-\mathbf {r'}) \theta _i(\textbf{r}) \theta _j(\mathbf {r'}), \end{aligned}$$

where the integral kernel $B(\textbf{r})$ is defined through the inverse of the elastic Green’s function. Hence, it is sufficient to perform the Fourier transform energy calculations for all pairs of the same martensite variant $s_i=s_j=1$ on the discrete lattice sites in the volume *V*; for periodic boundary conditions and identical grain shapes, it is sufficient to calculate the elastic interaction energy between a reference grain and all the other grains, due to translation invariance. In case of fixed average strain boundary conditions, an additional homogeneous term appears (see methods section), contributing both to the spin-spin interaction $J_{ij}$ as well as to the magnetic field term $h_i$, which is absent for zero average stress boundary conditions. The resulting fully populated matrix of coupling constants with both positive and negative entries has similarities to spin glass systems with random couplings, which have been investigated in the literature with conventional approaches, see e.g.^38^.

For the simplest case that the eigenstrain is purely dilatational and isotropic the Bitter-Crum theorem applies and the total energy depends only on the volume fraction of the martensite variant, where no interaction between the grains is present and only a self energy term remains^39^.

**Figure 2:**
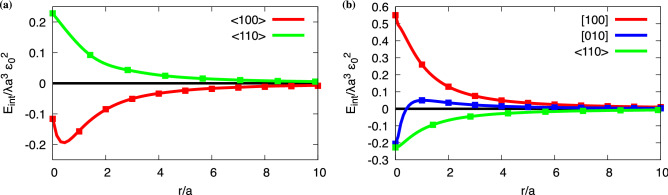
Interaction energies of two grains of equal variant type ($\mathbf {s_i=s_j}$). Interaction energies in the case of (**a**) shear eigenstrain and vanishing average stress and (**b**) tetragonal eigenstrain. The interaction energy per length is given in units of $\lambda a^3 \varepsilon _0^2$, and the computations were done using a system size of $L_x/a=L_y/a=50$, where *a* is the edge length of the grains. At distance $r/a=0$ the grains touch each other. The symbols on the continuous curves indicate the information for the interaction at discrete lattice sites, which is actually used in the annealer simulations.

For a nontrivial elastic interaction and the link to the previous 1D description, we consider a shear transformation strain with $\varepsilon _{xy}^0=\varepsilon _{yx}^0=\varepsilon _0$, where all other components vanish. In this case, we obtain a distance and orientation dependent interaction as depicted in Fig. 2a, which is computed here for the case of vanishing average stress, $\langle \sigma _{ik}\rangle =0$. Here and in the following parts the Poisson ratio is chosen as $\nu =1/4$ (i.e. $\lambda =\mu$).

The interaction energy is obtained by subtracting the elastic self energies $E_\text{self}$ for each of the two (isolated) martensite grains inside the austenitic matrix from the total elastic energy $E_\text{el}$ of the two-grain arrangement, i.e. $E_\text{int}=E_\text{el}-2E_\text{self}$, to normalize the interaction energy such that it decays to zero for large grain separations. For short distances, a transition between attraction and repulsion is found for the $\langle 100\rangle$ direction, whereas a purely repulsive interaction results for the diagonal $\langle 110\rangle$ directions. Due to the periodic boundary conditions, the result depends on the system size $V=L_x\times L_y$, as the grains also interact with their periodic images, hence $r\ll L_x, L_y$ is required to observe the decay of the interaction.

We note that in two dimensions the interaction energy decays asymptotically as $r^{-2}$, whereas in three dimensions it scales as $r^{-3}$ in large systems, which follows from the elastic Green’s function^40^. For the quantum annealer implementation, the interaction energies are needed only for the discrete lattice points (symbols on the curves). Although the decay of the elastic interaction may suggest to cut it off beyond a certain distance in real space, it turns out that such an approach is inappropriate, as it leads in the end to invalid equilibrium microstructures, and it is therefore essential to keep all interaction terms $J_{ij}$ with high precision to avoid spurious effects. We note that the formulation on the quantum annealer does not depend on the dimensionality, therefore the scaling plot in Fig. 1b applies here as well.

Based on the calculation of the elastic interactions, we obtain from the Ising model implementation on the quantum annealer with hybrid solver stripe patterns in $\langle 100\rangle$ direction as equilibrium structures. These patterns are irregular in the sense that the widths of the stripes are not uniform. This is in analogy to the 1D model, which was discussed above, where we found that the arrangement of the two variants is not determined. This coincidence, which is physically expected, is nontrivial from the model formulation, as (i) in the 1D model we had a distance independent interaction in the discretized model, where here the interaction is significantly more complex, but adds up to the same effective descriptions for the periodic arrangement; (ii) a rotation of the pattern by 90 degree is possible and sometimes obtained from the optimal configuration due to the discrete rotational symmetry; (iii) the fixing of the average stress compared to the given average strain in the 1D formulation can lead to unequal distributions of the different variants. In particular, for the presently considered absence of an external strain (implying a vanishing magnetic field in the Ising terminology), there is no constraint of the sort $\langle s_i\rangle = 0$ for the average spin alignment. All stripe configurations are energetically equivalent, which includes the possibility of a single variant configuration. These results therefore confirm simultaneously the accuracy of the elastic interaction calculation with the pairwise decomposition as well as the ability of the quantum annealer to identify the true ground state configurations.

**Figure 3:**
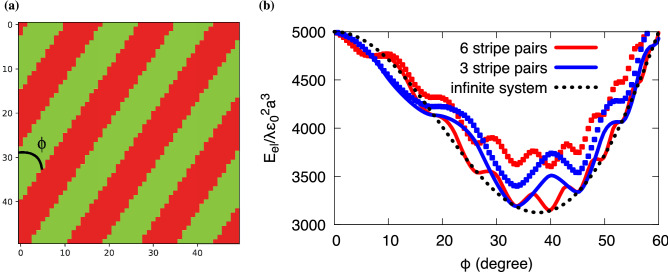
Resulting stripe patterns for tetragonal eigenstrain. (**a**) Equilibrium structure with three stripe pairs (counted along the horizontal axis) in a system consisting of $50\times 50$ cuboidal grains. A vanishing mean strain, $\langle \varepsilon _{ij}\rangle =0$, is imposed. The width of the stripes is uniform, consisting of grains with configuration $s_i=+1$ (red) and $s_i=-1$ (green). (**b**) Elastic energy of stripe patterns with different inclination angles $\phi .$ The solid curves correspond to smooth stripes (the grain size $a/L_x, a/L_y\ll 1$ is negligible) and show a pronounced stationary point for inclinations for which the pattern repeats periodically without kinks at the boundaries. The squares correspond to situations with the same number of stripes, where the system is discretized by $50\times 50$ quadratic grains, leading to pronounced aliasing effects, and the resulting elastic energy is higher than for the smooth stripes. This shifts the energetic minimum for 6 stripe pairs at $\phi \approx 40^\circ$ to a lower angle $\phi \approx 33^\circ$ with 3 stripe pairs. The infinite system size limit for smooth stripes is depicted as black dotted curve.

As next example, we use a tetragonal eigenstrain with the only nonvanishing components $\varepsilon _{xx}^0=-\varepsilon _{yy}^0=\varepsilon _{zz}^0=\varepsilon _0$. First, we consider again the situation with vanishing average stress, $\langle \sigma _{ij}\rangle = 0$. The corresponding interaction energy is shown in Fig. 2b for $\nu =1/4$. In this case, the equilibrium microstructure is trivial and consists of a single variant, as in this case the elastic energy is zero for the periodic system. Therefore, the situation differs from the previous shear transformation, where also lamellar arrangements with both variants lead to stress free situations. The reason is that any interface between two variants leads to a mismatch between adjacent variants for the tetragonal transformation, and therefore such a situation is energetically unfavorable here. A change of boundary conditions to vanishing average strain, $\langle \varepsilon _{ij}\rangle =0$, alters the situation, since then arrangements with equal amounts of both variants are preferred, as this lowers the volumic part of the elastic energy. In this case, we find regular inclined stripes as equilibrium pattern, as shown in Fig. 3a.

Again, the solution is not unique; in particular, due to translation invariance, the annealer returns also configurations where the stripes are shifted. Also, a switch of the sign of the inclination angle $\phi$ (see definition in the figure) leads to energetically equivalent solutions. However, we do not find ground state configurations which lead to different (absolute) inclination angles or strip widths or even irregular variations of the latter, contrary to the shear transformation case before.

The reason for the observed ground state morphologies is a combination of continuum elasticity effects, the granular structure of the material and constraints induced by periodic boundary conditions. Figure 3b shows the computed elastic energy for different numbers of regular arrangements of stripes in the periodic system as function of the inclination angle $\phi$. Here we see a pronounced influence of the grain size, as the elastic energy of configurations with regular stripe pairs with a discretization by $50\times 50$ grains (squares in the figure) is higher than for corresponding cases with very fine grains, where discretization effects do not play a role anymore (smooth curves). The oscillating nature is due to the periodic boundary conditions, as improper choices of the inclination angle lead to discontinuities of the stripe patterns at the boundaries, which is energetically unfavorable. Therefore, continuous patterns correspond to the stationary points of the curves. For specific angles, the curves for three and six stripe pairs meet at local minima, which is a consequence of the scale invariance of linear elasticity. From the smooth, continuum limit curves one would conclude that an angle of about $\phi \approx 40^\circ$ should lead to the energetically lowest configuration (absolute minimum of the smooth red curve). Moreover, in the limit of infinite systems, where periodic boundary conditions do not play a role anymore, an analytical treatment is possible, leading to the energy expression $E_\text{el}^\infty = V B(n)/2$ for equal volume fraction of the two variants with

$$B(n) = \frac{{4\mu }}{{\lambda + 2\mu }}\varepsilon _{0}^{2} {\text{ }}\left[ {(3\lambda + 2\mu ) - 2(3\lambda + 2\mu )n^{2} + 4(\lambda + \mu )n^{4} } \right]$$

with $n=\cos \phi$. Energy minimization gives the optimal angle $\phi =\cos ^{-1}\sqrt{5/8}\approx 37.8^\circ$, see Fig. 3b (minimum of the black dotted curve).

However, these predictions disagree with the finding from the quantum annealer, which favors a configuration with three stripe pairs at a lower angle of $\phi \approx 33^\circ$. This observation can be understood by consideration of the granular structure of the patterns investigated here, as the microstructure in the annealer simulations consists of $50\times 50$ square grains. First, the explicit appearance of the length scale *a* breaks the scale invariance of the periodic pattern, and therefore the minima of the energy curves belonging to the discrete microstructures (squares in Fig. 3b) do not coincide anymore at the local minima. Additionally, with increasing inclination antialiasing effects of the patterns become more relevant, and therefore the energy curves show an increasing disagreement with the continuum limit curves. As a result, the energetic minimum in the discrete microstructure indeed shifts toward a configuration with three stripe pairs at $\phi \approx 33^\circ$ (absolute minimum of the blue squares in Fig. 3b), which is in agreement with the prediction of the quantum annealer. Consequently, details of the granular structure can change the energetics compared to a full continuum approximation, especially since many local minima of the elastic energy are located close to each other.

### Variant selection in realistic microstructures

The approach presented above is not limited to mutually interacting cuboidal grains, but can also be applied to realistic microstructures. To illustrate the procedures, we have generated a microstructure consisting of $N=400$ grains using a Voronoi tesselation^41^. Each grain is allowed to take one out of two martensite variants with the tetragonal eigenstrain tensor, and we pre-compute all mutual elastic interactions between them. We note that contrary to the case with the cuboidal grains in a periodic array here we cannot exploit translational invarince due to the different shapes of the grains, and hence these elastic interaction energy calculations scale here as ${{\mathcal {O}}}(N^2)$ instead of ${{\mathcal {O}}}(N)$ before, although we still use periodic boundary conditions. Additionally, we consider now arbitrary given external strains $\langle \varepsilon _{ij}\rangle$, which leads to the appearance of a “magnetic” term like in the one dimensional description. With that, we can predict the equilibrium variant distribution within the microstructure using the hybrid quantum annealer, and this step is typically executed within a few seconds of runtime.

**Figure 4:**
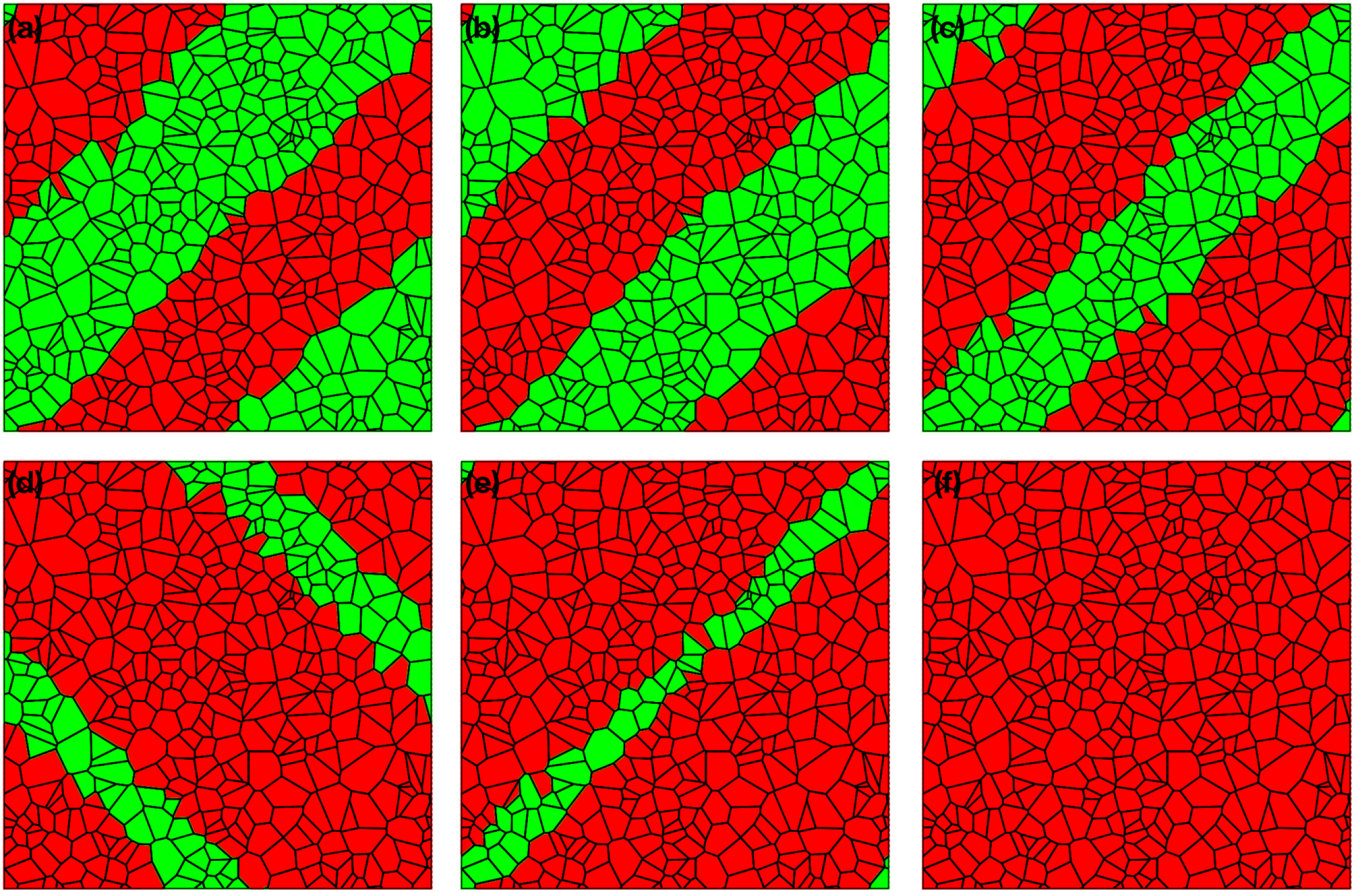
Resulting equilibrium variant distribution with uniform grain orientation. The microstructures consist of 400 grains and tensile strain is applied in horizontal (*x*) direction. Red (green) grains correspond to variant $s_i=+1$ ($s_i=-1$). The tensile strain is (**a**) $\langle \varepsilon _{xx}\rangle /\varepsilon _0 = 0$, (**b**) $\langle \varepsilon _{xx}\rangle /\varepsilon _0 = 0.1$, (**c**) $\langle \varepsilon _{xx}\rangle /\varepsilon _0 = 0.5$, (**d**) $\langle \varepsilon _{xx}\rangle /\varepsilon _0 = 0.9$, (**e**) $\langle \varepsilon _{xx}\rangle /\varepsilon _0 = 1.1$ and (**f**) $\langle \varepsilon _{xx}\rangle /\varepsilon _0 = 1.3$.

Examples for the equilibrium microstructures are shown in Fig. 4 as function of the externally applied strain $\langle \varepsilon _{xx}\rangle$, whereas the other average strain components vanish.

The observed microstructures are indeed similar to what we have found before using the square discretization, although here the band widths and orientation deviate from the previous case due to microstructural details and the smaller number of grains, and these effects can be explained using an analysis similar to the one done for Fig. 3b. We note that in these microstructures all grains have the same orientation, and therefore the application of a tensile strain strongly favors the selection of the grain variant $s_i=+1$ (for a compressive situation we observe the opposite behavior), and we find a full alignment of all variants in the last snapshot.

**Figure 5:**
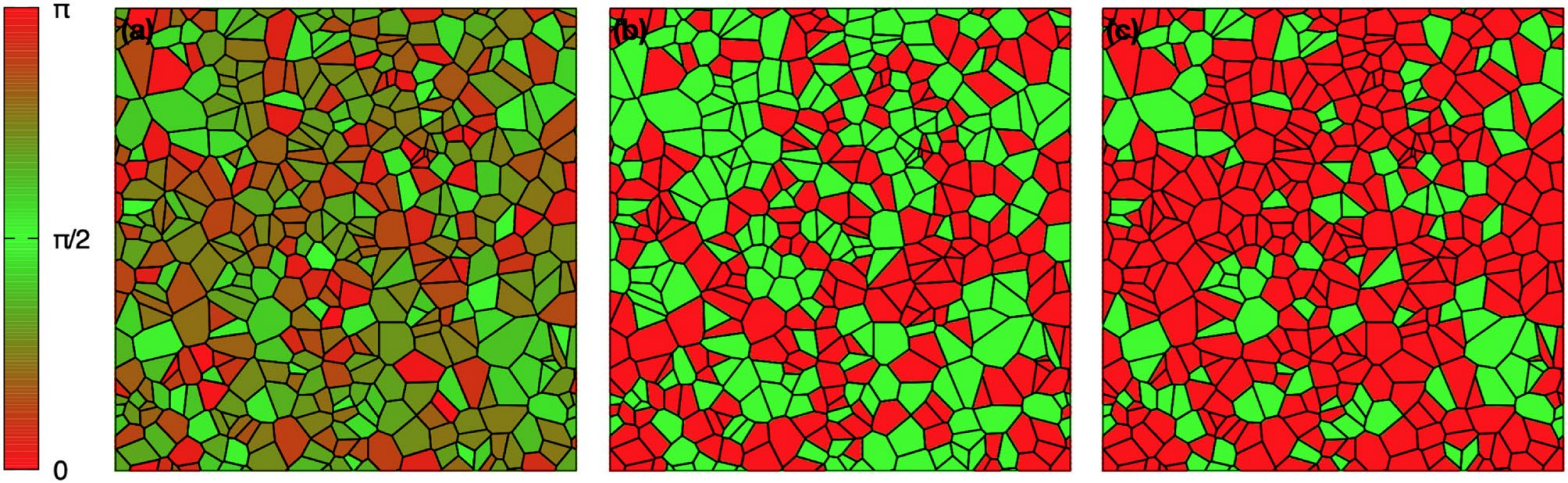
Resulting equilibrium variant distribution with random grain orientation. (**a**) Grain orientation map corresponding to the microstructures. In the color bar the grain rotation angle is given in radian (modulo $\pi$ due to symmetry). The rotation axis is along the [001] direction. The microstructures consist of 400 grains and tensile strain is applied in horizontal (*x*) direction. The grains have a random orientation, which is the same for all cases, based on a uniform distribution. The tensile strain in horizontal direction is (**b**) $\langle \varepsilon _{xx}\rangle /\varepsilon _0 = 0$ and (**c**) $\langle \varepsilon _{xx}\rangle /\varepsilon _0 = 2.1$. Red (green) grains correspond to variant $s_i=+1$ ($s_i=-1$).

Additionally, we have performed the same analysis for grains with uniformly distributed random orientation, which implies a rotation of the local transformation strain tensor, see Fig. 5 for the grain orientations and for the variant selection.

Here, also the equilibrated spatial distribution of the variants appears to be irregular. Application of a tensile strain again favors the “alignment” of the variant, but this time even for high strains not all grains select the same variant, which is due to the local rotation. In fact, a grain, which is rotated by $90^\circ$ with respect to the straining direction has a preference to be in variant state $s_i=-1$, as then the direction of expansion is aligned with the external tensile strain. This can be clearly seen e.g. in Fig. 5(c) for the highest tensile strain in *x* direction, where the remaining patches with “spin” $s_i=-1$ correspond to the grains with orientation close to $\pi /2$ (or $3\pi /2$). We emphasize that for a given microstructure (shapes of all grains) the mutual elastic grain-grain interactions have to be computed only once. As mentioned before, this step has to be done with high precision, and consequently this is the step which demands the highest amount of computing time. After that, all changes of the external boundary conditions affect only the $k=0$ mode contributing to the interactions $J_{ij}$ and $h_i$, and these terms can be calculated analytically (see methods section). As each hybrid quantum annealing calculation typically requires only a few seconds, the entire microstructural change during mechanical loading can be calculated extremely fast.

## Discussion

The central result of the present paper is the shown optimization of microstructures via quantum annealing, exhibiting a clear performance advantage of the novel approach compared to conventional energy minimization strategies. The brute force approach is not recommended, whereas optimized simulated annealing algorithms produce good results. However, quantum annealing represents the by far fastest method for optimization problems, particularly for systems with high numbers of grains (spins) and non-vanishing coupling constants and biases, and allows the determination of ground state configurations for system sizes, which are not accessible for the classical algorithms on reasonable computing timescales.

For a system consisting of *N* grains, we need to compute ${{\mathcal {O}}}(N^2)$ spin-spin interactions. These elastic energy calculations have to be done with high accuracy, and therefore they dominate the overall computing time. After that, ${{\mathcal {O}}}(2^N)$ spin configurations need to be compared to identify the equilibrium configuration. For the conventional algorithm, this combinatorial step dominates the total computational effort already for low values of *N*. In contrast, with the quantum annealer or its hybrid variant the computation time for the minimization of the Ising energy expression is completely negligible compared to the elastic interaction energy computations. Hence, we have demonstrated that QA is able to drastically optimize the search for microstructural equilibrium states in solid phases with long-range elastic interactions. Already today, the usage of hybrid quantum annealing enables the computation of microstructures with several thousand grains which all interact with each other, which is essential for a realistic modeling of microstructures inside various materials.

For many application relevant investigations, it is critical to understand whether and how models can be formulated that they are suitable for quantum computing. We have demonstrated this here for the particular case of long-range elastic interactions. Extensions towards the consideration of interfacial energy, multiple martensite variants, anisotropic elasticity, orientation relationships between grains and phases, and different spatial dimensions are obvious, as they do not conceptually influence the presented strategy of formulating the problem in terms of an Ising model. Inhomogeneous elasticity and the proximity to free surfaces can effectively lead to many-body interactions, for which perturbative extensions or the introduction of product spin variables are promising directions^37,42^. Beyond the purely elastic effects, further potential applications comprise phase changes in multi-phase solid state batteries, phase transformations in high strength steels or other materials like ferroelectrics. Overall, the separation of continuous and discrete degrees of freedom and the quantum treatment of the latter may also be beneficial for hybrid phase field and quantum annealing descriptions which combine a variant selection with a grain morphology evolution in an efficient way to drastically reduce computing time demands of existing approaches for application relevant sample sizes.

## Methods

### Quantum annealing

Like general purpose quantum computers, a quantum annealer is built from qubits, which here store and process information using superconducting loops. A clockwise or anticlockwise circulating current in such a loop represents different spin states^12^. In each qubit superconducting loops interact with external flux biases, which allows to construct an energy landscape, where the fluxes influence barrier height and energy difference^12^. At the start of the computation, the system is initialized in the ground state of a known Hamiltonian $H_0\sim -\sum _i \sigma _i^x$ with Pauli matrices $\sigma _i$, i.e. a strong transverse magnetic field^13,43^. During the annealing process, the Hamiltonian is turned into the desired one based on an Ising model^11^ $H_p = \sum _ih_is_i+\sum _{i<j}J_{ij}s_is_j$ with spin states $s_i=\pm 1$, bias $h_i$ and coupling strength $J_{ij}$ between qubits *i* and *j*, for which an energetic minimum is sought, $\min _{\{s_i=\pm 1\}} H_p$. Both Hamiltonians do not commute^11^, and the time of the initial Hamiltonian to adopt the low energy state is sufficiently large to ensure the validity of the adiabatic theorem of quantum mechanics^44^, which states that a system remains in its eigenstate, if changes occur adiabatically. Notice that the quantum annealing employs tunneling to leave metastable regions, contrary to the simulated annealing^6^. Another important quantum mechanical principle in quantum annealing is the entanglement and the usage of entangled states inside quantum annealing processors (QPU)^45^.

As in practise this approach does not always deliver the lowest energy state, especially if energetically close low energy states exist, a suitable number of repetitions is made and the configuration with the lowest detected energy is taken. If the Ising problems do not match the architecture of the QPU, a subgraph of coupled qubits, know as chains, cover one variable of the problem in the so called minor embedding^36,46^. Additionally, for huge systems hybrid quantum annealing exploits classical algorithms and the interplay with quantum annealing in areas of high computational demands using a QPU coprocessor working with generic parameters for up to 11616 spin variables on the D-Wave Advantage system^36,47^. In practise, the D-Wave framework Leap^48^ allows a direct formulation in terms of a problem Ising Hamiltonian.

### Brute force minimization

For *N* spins we compute the energy of all $2^N$ possible configurations to determine the minimum. This deterministic approach delivers the true ground state energy but has a high computational effort.

### Simulated annealing

For this probabilistic approach^49^ a random initial configuration is chosen. A new candidate configuration, which we generate here by a single spin flip, is accepted if its energy is lower than the previous value. If the energy is higher by an amount $\Delta E$, the configuration is accepted with a probability given by the Boltzmann factor $\exp (-\Delta E/T)$, in order not to get stuck in local energy minima. During the simulation, the temperature *T* is reduced according to a specific cooling strategy, in order to converge towards an energetic minimum at the end of the simulation. As our main goal is not to maximize the performance of the (classical) algorithms but rather to demonstrate the general scaling behavior, we refrain from a detailed discussion of the parameter optimization of the probabilistic simulated annealing approach. This includes in particular the use of suitable stopping criteria when no further reduction of the energy occur, as well as the use of problem adapted cooling strategies. For the simulated annealing approach we use single spin flips trials in each iteration, and the temperature *T* is decreased each time by $\Delta T/\mu _\text{eff}\varepsilon _0^2d ^2 = 10^{-6}$, which delivers a good performance for large system sizes. The simulations are stopped after a fixed number of $10^{7}$ steps, which is optimized for the largest considered spin system with $N=150$ in Fig. 1b, leading to a scaling of the computation time $\sim N^2$ due to the calculation of the interaction energy.

### Elasticity

We solve the elastic problem of a multi-grain setup with homogeneous linear elasticity, i.e. all variants and phases are assumed to have the same elastic constants. Also, coherent interfaces are assumed, which means continuity of displacements at the interfaces. The martensite variants have different stress free strains (or eigenstrains) compared to the mother austenite phase, hence the stress-strain relation reads for general linear elasticity $\sigma _{ij} = \lambda _{ijkl}(\varepsilon _{kl}-\varepsilon _{kl}^{(0)})$, where $\varepsilon _{kl}^{(0)}(\textbf{r})$ is the local stress free strain tensor and $\lambda _{ijkl}$ the elastic tensor. We determine the elastic equilibrium configuration, which obeys the condition $\partial \sigma _{ij}/\partial x_j=0$ in bulk domains and the continuity of normal stresses at interfaces, using Fourier transformation approaches^32^. From that, the elastic energy can be computed in reciprocal space as^32^

3 $$\begin{aligned} E_\text{el} = \frac{V}{2} \sum _{\text{k}\ne 0} B(\textbf{n}) |\hat{\theta }(\textbf{k})|^2 \end{aligned}$$

for a periodic system with vanishing average stress as boundary condition, where $\hat{\theta }(\textbf{k})$ is the Fourier transform of the indicator field $\theta (\textbf{r})$ and $B(\textbf{n})$ with $\textbf{n}=\textbf{k}/k$ equals $B(\textbf{n}) = \sigma _{ij}^0\varepsilon _{ij}^0 - n_i \sigma _{ij}^0 \Omega _{jk} \sigma _{kl}^0 n_l$ with $\sigma ^0_{ij}=\lambda _{ijkl}\varepsilon _{kl}^0$. Here, $\Omega _{ij}(\textbf{n})$ is the normalized Green tensor for displacements, which is defined through its inverse as $\Omega _{ik}^{-1} = \lambda _{ijkl}n_j n_l$. The summation in Eq. (3) is over discrete vectors due to the periodic boundary conditions in real space. The summation is in principle infinite, and can be efficiently computed using the decoration technique^50^. For average strain boundary conditions, i.e. a prescribed value of $\langle \varepsilon _{ij}\rangle$, an additional homogeneous ($\textbf{k}=0$) contribution appears in Eq. (3), which reads $E_\text{hom} = V \lambda _{ijkl} (\langle \varepsilon _{ij}\rangle - \langle \varepsilon _{ij}^{(0)}\rangle ) (\langle \varepsilon _{kl}\rangle - \langle \varepsilon _{kl}^{(0)}\rangle )/2$, which can be calculated analytically.

## Data Availability

Data that was obtained during this project will be made available by the corresponding author upon request.
